# Investigation of the Relationship Between Chronic Stress and Insulin Resistance in a Chinese Population

**DOI:** 10.2188/jea.JE20150183

**Published:** 2016-07-05

**Authors:** Yu-Xiang Yan, Huan-Bo Xiao, Si-Si Wang, Jing Zhao, Yan He, Wei Wang, Jing Dong

**Affiliations:** 1 Department of Epidemiology and Biostatistics, School of Public Health, Capital Medical University, Beijing, China; 2 Municipal Key Laboratory of Clinical Epidemiology, Beijing, China; 3 Department of Preventive Medicine, Yanjing Medical College, Capital Medical University, Beijing, China; 4 Health Medical Examination Center, Xuanwu Hospital, Capital Medical University, Beijing, China; 5 School of Medical Sciences, Edith Cowan University, Perth, Australia

**Keywords:** stress, insulin resistance, cortisol, HOMA-IR

## Abstract

**Background:**

Chronic stress may facilitate the development of metabolic diseases. Insulin resistance is present long before the clinical manifestations of individual metabolic abnormalities. To explore whether chronic stress is an independent risk factor of insulin resistance, we investigated the relationship between the stress system, selected parameters of energy homeostasis, and insulin resistance in a Chinese population.

**Methods:**

We recruited 766 workers employed at four companies in Beijing. The degree of insulin resistance was determined using the homeostasis model assessment of insulin resistance (HOMA-IR). The highest quartile of HOMA-IR among all study subjects was further defined as insulin resistance in our study. The short standard version of the Copenhagen Psychosocial Questionnaire (COPSOQ) was used to assess job-related psychosocial stress. Pearson’s correlation coefficients were calculated between cortisol level and HOMA-IR and components of metabolic syndrome, with stratification by gender. The relationship between cortisol and HOMA-IR independent of obesity was analyzed using a linear mixed model with company as a cluster unit.

**Results:**

The values of the two scales of COPSOQ, including “demands at work” and “insecurity at work”, were significantly associated with insulin resistance and cortisol concentration (*P* < 0.05). Cortisol was significantly positively correlated with glucose, HOMA-IR, and waist circumference in males and females (*P* < 0.05). After adjusting for potential confounders, cortisol was an independent positive predictor for HOMA-IR (*P* < 0.05).

**Conclusions:**

These findings showed that chronic stress was associated with insulin resistance and may contribute to the development of insulin resistance.

## INTRODUCTION

Insulin resistance (IR) is a state in which physiologic concentrations of insulin produce a subnormal biologic response.^1^ IR is directly related to the risk of developing metabolism-related disorders, including type 2 diabetes mellitus (T2DM), cardiovascular disease, and even cancer.^2^ China has experienced an epidemic of major chronic diseases, such as stroke, coronary heart disease, cancer, diabetes, and hypertension, in recent decades. In addition to genetic causes, environmental influences, such as chronic stress, behavioral disturbances, and dietary deficiency, have now emerged as contributors to the development of metabolic diseases.^3^ With urbanization, industrialization, and strong economic growth in China, psychosocial stress is highly prevalent in modern society. Psychological stress can affect health through complex interactions among neuroendocrine responses and energy homeostasis.^3^ One of the major neuroendocrine systems responding to psychological stress is the hypothalamus-pituitary-adrenal (HPA) axis, with cortisol secretion as its final hormonal effector. As an indicator of HPA activity, cortisol is an important hormonal signal of chronic stress response.^4^ Elevated cortisol level is hypothesized to induce visceral fat accumulation and stimulate gluconeogenesis and lipolysis pathways,^5^ which can result in IR.

Preclinical status of diseases and their early detection have become major issues in the promotion of the basic health service in the reform of health care in China.^6^ IR is present long before the clinical manifestations of individual metabolic abnormalities. The high prevalence of IR and its association with dyslipidemia, hypertension, hyperinsulinemia, and high coronary and cerebrovascular mortality put it in the forefront as a plausible target for aggressive intervention.^7^ Although chronic stress may be associated with progression or development of IR, little evidence exists in the Chinese population. To explore whether chronic stress is an independent risk factor of IR, we investigated the relationship between the stress system, selected parameters of energy homeostasis, and IR in a Chinese population.

## MATERIALS AND METHODS

### Subjects

A cross-sectional study was conducted among staff who received routine annual physical examinations (for at least 2 consecutive years) from March to June 2014 at the health examination center of Xuanwu Hospital, Capital Medical University. The subjects were required to be aged from 40 to 60 years. We excluded those who had a history of drug treatment for elevated plasma glucose, drug treatment for dyslipidemia, or recent use of steroid medication. Subjects were also excluded from the study if they had acute inflammatory diseases, hepatic or renal failure, cardiac failure, mood disorders, autoimmune disease, or cancer.

The sample size was calculated with power of 0.9 and Pearson’s correlation coefficient (*r*) of 0.18 between cortisol and HOMA-IR in the pre-survey, which yielded a sample size of 320 male and 320 female subjects (PASS 11.0). The calculated sample size was increased by an additional 10% to account for the random cluster sampling, resulting in a total sample size of 704 participants. According to the medical records in 2013, there were 2693 eligible subjects from all 13 companies whose workers participated in annual physical examinations during March to June. Four companies randomly selected from these 13 were used for cluster sampling. Ultimately, 766 subjects who signed informed consent forms were enrolled. Both the university and hospital ethics committees approved the study.

### Data collection

A structured questionnaire was used to collect information on demographic data, environmental exposure history, and medical and family histories. Current cigarette smokers were defined as those who smoked ≥1 cigarette/day. Alcohol use was defined as intake of wine/beer/cider/spirits ≥1 time per week. Physical activity was defined as walking or riding ≥15 min/day, doing sports or physical exercise >2 h/week, or lifting or carrying heavy objects at work daily.^8^

The Chinese translation and adaptation of the short standard version of the Copenhagen Psychosocial Questionnaire (COPSOQ) was used to assess job-related psychosocial stress.^9^^–^^11^ The COPSOQ version used in this survey comprises 5 scales including 34 items concerning (1) demands at work, (2) work organization and content, (3) interpersonal relations and leadership, (4) insecurity at work, and (5) job satisfaction. All scales had a 5-point Likert format (except scales measuring insecurity at work), reflecting either intensity or frequency. These scales were transformed into a theoretical range, extending from 0 (do not agree at all) to 100 (fully agree) points. The higher score for scales of “demands at work” and “insecurity at work” and lower score for scales of “work organization and content”, “interpersonal relations and leadership”, and “job satisfaction” indicate higher levels of job strain.

### Measurement of anthropometric parameters

Body height and weight were measured with subjects wearing light clothing without shoes. Body mass index (BMI) was calculated as weight in kilograms divided by height in meters squared (kg/m^2^). Waist circumference was measured to the nearest 1.0 cm at the height of the navel upon breath intake using a non-extendable linen measure. Blood pressure was measured in the right arm using a standard mercury sphygmomanometer after 5 minutes of rest with each participant seated. The average value of three consecutive measurements was used in the analyses.

### Laboratory examination

Following an overnight fast, venous blood samples were collected between 7:30 and 8:30 a.m. in a calm state. All samples were immediately centrifuged for laboratory measurements. Total cholesterol (TC), high-density lipoprotein-cholesterol (HDL-C) and triglycerides (TG) were measured using standard laboratory methods (Hitachi autoanalyzer 7060; Hitachi, Tokyo, Japan). Low density lipoprotein-cholesterol (LDL-C) was calculated using the Friedewald method. Fasting serum glucose levels were measured by the glucose oxidase method. Serum cortisol and insulin were evaluated using radioimmunoassay with a γ radioimmunoassay counter (GC-911) (USTC, Chuangxin, China). The intra-assay coefficient of variation were <5.5% and <10.0% for the assays.

The homeostasis model assessment for insulin resistance (HOMA-IR) was calculated as (insulin [uIU/mL] × glucose [mmol/L])/22.5.^12^ The highest quartile of HOMA-IR among all study subjects was defined as IR in our study.

### Statistical analysis

Population demographic and clinical characteristics were described using means and standard deviations (SDs) for continuous variables and proportions for categorical variables. Differences in characteristics between subgroups according to cortisol level were examined using Independent Samples T-tests for continuous variables and Pearson Chi-Square tests for categorical variables. Bivariate Pearson’s correlation coefficients were calculated between cortisol, insulin, HOMA-IR, and components of metabolic syndrome, with stratification by gender. Partial correlation analysis was performed to determine the association between psychosocial stress (5 scales of COPSOQ) and HOMA-IR after adjustment for cortisol level. The relationship between cortisol and HOMA-IR independent of obesity was analyzed using a linear mixed model with HOMA-IR as a dependent variable and cortisol as an independent variable, with adjustment for age, smoking, drinking, and physical exercise. To control the effect of clustering of cases within companies, a ‘company’ variable was defined as a subject grouping variable in the model. All statistical analyses were conducted with the SPSS statistical package for Windows version 17.0 (SPSS Inc., Chicago, IL, USA). A threshold of *P* < 0.05 was considered significant.

## RESULTS

There were 335 males (43.73%) and 431 females (56.27%) in this study (Table 1). The mean (SD) age of the sample was 46.98 (6.60) years. Most (86.03%) of the participants were white-collar workers. The 766 subjects were divided into two groups by the highest quartile of HOMA-IR (2.37): an IR group (HOMA-IR ≥2.37) and a non-IR group (HOMA-IR <2.37).

**Table 1.** Demographic and clinical characteristics of 766 subjects and the two subgroups stratified according to the cortisol median

Variable	Total	M1	M2	*t*	*P*
Age, years	46.98 (6.60)	46.48 (6.43)	47.47 (6.73)	2.080	0.038
Gender, male/female	335/431	152/231	183/200	5.098^a^	0.024
WC, cm	80.31 (10.24)	79.28 (9.63)	81.34 (10.73)	2.796	0.005
BMI, kg/m^2^	24.27 (3.03)	24.10 (3.02)	24.44 (3.03)	1.553	0.121
SBP, mm Hg	119.62 (14.60)	118.74 (14.95)	120.49 (14.22)	1.660	0.097
DBP, mm Hg	76.81 (10.38)	76.31 (10.57)	77.32 (10.17)	1.348	0.178
Glucose, mmol/L	5.35 (0.90)	5.23 (0.78)	5.48 (0.99)	3.911	<0.001
TC, mmol/L	4.79 (0.90)	4.66 (0.88)	4.92 (0.89)	4.142	<0.001
TG, mmol/L	1.31 (1.00)	1.27 (0.98)	1.36 (1.01)	1.171	0.242
HDL-C, mmol/L	1.60 (0.58)	1.59 (0.53)	1.62 (0.62)	0.521	0.603
LDL-C, mmol/L	2.67 (0.87)	2.58 (0.84)	2.76 (0.90)	2.892	0.004
Insulin, uIU/mL	8.06 (2.98)	7.64 (2.76)	8.48 (3.13)	3.921	<0.001
HOMA-IR	1.94 (0.87)	1.81 (0.82)	2.08 (0.91)	4.372	<0.001
Cortisol, ng/mL	186.03 (33.93)	161.75 (14.86)	210.30 (30.04)	28.35	<0.001
Physical activity, *n* (%)	508 (66.3)	258 (67.4)	250 (65.3)	0.374^a^	0.541
Smoking, *n* (%)	83 (10.8)	38 (9.9)	45 (11.8)	0.662^a^	0.416
Alcohol use, *n* (%)	161 (42.0)	80 (20.9)	81 (21.2)	0.008^a^	0.929

Values are reported as mean (standard deviation), unless otherwise noted.

DBP, diastolic blood pressure; HDL-C, high-density lipoprotein cholesterol; HOMA-IR, homeostasis model assessment of insulin resistance; LDL-C, low-density lipoprotein cholesterol; SBP, systolic blood pressure; TC, total cholesterol; TG, triglycerides; WC, waist circumference.

^a^χ^2^ value.

Table 1 presents demographic and clinical characteristics of the two subgroups stratified according to the median cortisol level into lower (M1) and higher (M2) groups. Age and gender were significantly correlated with cortisol level (*P* < 0.05). Males had significantly higher cortisol concentrations compared to females (191.14 [SD, 37.94] vs 182.06 [SD, 29.89], *P* < 0.001). No significant differences were observed for smoking, drinking, and physical exercise distribution across groups (*P* > 0.05). Waist circumference, glucose, TC, LDL-C, insulin, and HOMA-IR were significantly higher in M2 than in M1. Distribution of BMI, blood pressure, TG, and HDL-C showed no difference between the two groups (*P* > 0.05).

The Cronbach’s α coefficient of the COPSOQ was 0.851, indicating good internal consistency. Overall, the IR subjects had a higher level of psychosocial stress than non-IR subjects (Table 2). Compared to the non-IR group, the mean values of scales on “demands at work” and “insecurity at work” were significantly higher in the IR group than in the non-IR group (*P* < 0.001). The results also indicated that staff who reported that they were highly stressed from their job often showed a higher level of HOMA-IR (Table 3). Significant association between COPSOQ scales of “demands at work” and “insecurity at work” and cortisol levels confirmed an interaction between psychosocial stress and HPA activity (Table 3). After adjustment for the influence of cortisol, HOMA-IR was also significantly associated with scales of “demands at work” (partial *r* = 0.149, *P* < 0.001) and “insecurity at work” (partial *r* = 0.117, *P* = 0.001).

**Table 2.** Association between psychosocial stress and IR

Variables	IR group (*n* = 194)	non-IR group (*n* = 572)	*F*^a^	*P*
Demands at work	58.53 (17.84)	50.07 (17.01)	33.19	<0.001
Work organization and content	61.03 (17.51)	61.33 (17.52)	0.026	0.612
Interpersonal relations and leadership	60.04 (19.60)	60.92 (19.13)	0.014	0.901
Insecurity at work	52.71 (41.77)	39.82 (40.55)	13.23	<0.001
Job satisfaction	56.60 (22.32)	59.89 (21.91)	1.947	0.163

IR, insulin resistance.

Values reported as mean (standard deviation).

^a^Adjusted for age, gender, company, smoking, drinking and physical exercise.

**Table 3.** Pearson’s correlation matrix of psychosocial stress, cortisol and HOMA-IR

Scales	1.	2.	3.	4.	5.	6.	7.
1. Cortisol	1.00						
2. HOMA-IR	0.20	1.00					
3. Demands at work	0.17^b^	0.18^b^	1.00				
4. Work organization and content	0.03	0.00	−0.08^a^	1.00			
5. Interpersonal relations and leadership	−0.01	0.00	0.05	0.53^b^	1.00		
6. Insecurity at work	0.15^b^	0.14^b^	0.42^b^	0.02	−0.04	1.00	
7. Job satisfaction	0.01	−0.03	−0.19^b^	0.40^b^	0.54^b^	−0.15^b^	1.00

HOMA-IR, homeostasis model assessment of insulin resistance.

^a^*P* < 0.05.

^b^*P* < 0.01.

Pearson’s correlation coefficients of cortisol with HOMA-IR and parameters of energy homeostasis are presented in Table 4. Cortisol concentrations were significantly positively correlated with glucose and insulin concentrations and HOMA-IR in males and females (*P* < 0.05). To explore the relationship between cortisol and abdominal obesity, Pearson correlations of cortisol with BMI and waist circumference were analyzed. Cortisol was significantly correlated with waist circumference in male and female subjects (*P* < 0.05). Pearson correlation between cortisol and components of metabolic syndrome, including SBP, DBP, TG, and HDL-C showed that cortisol was significantly positively correlated with SBP and TG in female subjects (*P* < 0.05).

**Table 4.** Pearson’s correlation coefficients of cortisol with parameters of energy homeostasis

Variables	Male (*n* = 335)		Female (*n* = 431)	
	
	*r*	*P*	*r*	*P*
Glucose	0.202	<0.001	0.172	<0.001
Insulin	0.127	0.020	0.177	<0.001
HOMA-IR	0.178	0.001	0.190	<0.001
TC	0.187	0.001	0.237	<0.000
TG	−0.003	0.954	0.132	0.006
HDL-C	−0.014	0.798	0.023	0.640
LDL-C	0.131	0.016	0.168	0.000
WC	0.131	0.016	0.127	0.008
BMI	0.094	0.086	0.069	0.155
SBP	0.050	0.365	0.149	0.002
DBP	0.028	0.604	0.054	0.262

BMI, body mass index; DBP, diastolic blood pressure; HOMA-IR, homeostasis model assessment of insulin resistance; HDL-C, high-density lipoprotein cholesterol; LDL-C, low-density lipoprotein cholesterol; SBP, systolic blood pressure; TC, total cholesterol; TG, triglycerides; WC, waist circumference.

To explore the relationship between cortisol and IR independent of obesity, linear mixed models were used among male and female subjects, with company as a subject-grouping variable (Table 5 and Table 6). The following categories including components of metabolic syndrome were also analyzed as independent variables: serum TG, HDL-C, SBP, DBP, and waist circumference or BMI. Possible confounders, including age, smoking, drinking, and physical exercise were adjusted in the linear mixed models.

**Table 5.** Linear mixed model analysis of the relationship between cortisol and HOMA-IR (adjusted for WC)

Variables	Male (*n* = 335)		Female (*n* = 431)	
	
	β coefficient	*P*	β coefficient	*P*
Age	0.009	0.268	0.006	0.290
Smoking	0.047	0.705	0.448	0.044
Drinking	0.036	0.768	0.069	0.497
Physical exercise	−0.014	0.890	−0.110	0.273
Cortisol	0.003	0.008	0.004	0.002
TG	0.027	0.610	0.135	0.001
HDL-C	−0.059	0.436	0.151	0.047
SBP	0.011	0.047	−0.004	0.341
DBP	−0.007	0.325	0.002	0.714
WC	0.013	0.018	0.017	0.002

DBP, diastolic blood pressure; HDL-C, high-density lipoprotein cholesterol; SBP, systolic blood pressure; TG, triglycerides; WC, waist circumference.

**Table 6.** Linear mixed model analysis of the relationship between cortisol and HOMA-IR (adjusted for BMI)

Variables	Male (*n* = 335)		Female (*n* = 431)	
	
	β coefficient	*P*	β coefficient	*P*
Age	0.009	0.274	0.006	0.284
Smoking	0.078	0.525	0.435	0.005
Drinking	0.021	0.862	0.065	0.520
Physical exercise	−0.026	0.802	−0.112	0.176
Cortisol	0.004	0.006	0.004	0.001
TG	0.023	0.667	0.129	0.001
HDL-C	−0.063	0.405	0.149	0.049
SBP	0.012	0.029	−0.003	0.433
DBP	−0.009	0.265	0.001	0.913
BMI	0.041	0.017	0.049	0.001

BMI, body mass index; DBP, diastolic blood pressure; HDL-C, high-density lipoprotein cholesterol; SBP, systolic blood pressure; TG, triglycerides.

For males, cortisol was a significant positive predictor of HOMA-IR (β coefficient = 0.003; *P* = 0.008), independent of components of metabolic syndrome, including waist circumference. If waist circumference was replaced by BMI among the above independent variables in the linear mixed model, cortisol was also independently associated with HOMA-IR (β coefficient = 0.004, *P* = 0.006).

For females cortisol was a significant positive predictor for HOMA-IR (β coefficient = 0.004; *P* = 0.002), independent of components of metabolic syndrome, including waist circumference. If waist circumference was replaced by BMI among the above independent variables in the linear mixed model, cortisol was also independently associated with HOMA-IR (β coefficient = 0.004, *P* = 0.001).

## DISCUSSION

The present study examined the relationships between cortisol and related parameters of IR among employees at four companies in Beijing, China. Our results suggested that a higher degree of chronic psychosocial stress was associated with IR. In males and females, cortisol levels were positively associated with insulin, glucose, and HOMA-IR. Linear mixed models showed that IR was consistently associated with cortisol independent of abdominal obesity. Although methodological limitations of cross-sectional research need to be considered, these results support the hypothesis that stress might be involved in pathways leading to IR through disruptive interference between the stress system and mechanisms of energy homeostasis.

Growing evidence indicates that psychosocial factors play some role in the etiology and progression of visceral adiposity, metabolic syndrome, and T2DM. For example, work stress and low emotional support in women and sleeping disorders in men are stress factors that have been associated with development of T2DM.^13^ The possible pathophysiological mechanisms for the association between chronic psychological stress and IR or diabetes involve hyper-stimulation of the HPA axis due to stress.^14^ In this study, the valid and relevant short version of the COPSOQ instrument was used to assess psychosocial stress at work. This instrument covers the broad construct of work-related psychological factors using a multidimensional concept that includes seven major theories in occupational health psychology.^9^ Significantly higher levels of “demands at work” and “insecurity at work” were observed among IR subjects than non-IR subjects, suggesting that psychosocial stress may contribute to the development of IR. The significant association observed between COPSOQ and cortisol in the present study supports the concept that, as a stimulus, psychological stress activates the HPA and results in a physiological change or adaption.

According to Eller et al, cortisol levels increase in situations such as work stress and unemployment,^15^ and this is expected to lead to accumulation of abdominal fat. Our study showed that serum cortisol is positively correlated with waist circumference among male and female subjects, supporting findings that repeated or chronic stress plays a potential role in the development of overweight and obesity.^5^

The positive relationship between cortisol and insulin in the present study corresponds with previously reported effects of dexamethasone administration on IR,^16^ compensated by an increase in insulin secretion and elevated plasma concentrations.^17^ The metabolic effects of cortisol are largely explained by its ability to antagonize the actions of insulin.^18^ Cortisol impairs glucose uptake in peripheral tissues like fat and muscle, and this can involve impaired translocation to the plasma membrane of glucose transporter 4.^14^ Cortisol might also influence glucose metabolism, possibly by stimulating gluconeogenesis in the liver, leading to increased plasma glucose.^19^ This phenomenon may be caused by cortisol enhancing expression of gluconeogenic enzymes (eg, phosphoenolpyruvate carboxykinase and glucose-6-phosphatase).^20^ Some of the insulin-antagonistic effects, both at the liver and in skeletal muscle, may be secondary to a lipolytic effect exerted by glucocorticoids.^18^^,^^21^ We also observed significant correlation of cortisol with TC and LDL-C in this study.

To our knowledge, this is the first large-scale epidemiological study to investigate the relationship between morning serum cortisol concentrations and parameters of IR in a Chinese population. The findings of this study support the hypothesis that stress might facilitate IR by modulating the stress system and mechanisms of energy homeostasis. However, there are several limitations in our study. A single-point determination of cortisol is not sufficient to be considered a definitive measure of HPA axis activity. Furthermore, the association of chronic stress with IR in a cross-sectional study does not prove causality; longitudinal cohort studies are needed to confirm our findings.

### Conclusions

Our findings showed that chronic stress was associated with IR and may contribute to the development of IR. Chronic stress may emerge as a new target for prevention of IR.

## References

[1] Caro JF. Clinical review 26: Insulin resistance in obese and nonobese man. J Clin Endocrinol Metab. 1991;73:691–5. 10.1210/jcem-73-4-691 1890146

[2] Li L, Guo Y, Chen Z, Chen J, Peto R. Epidemiology and the control of disease in China, with emphasis on the Chinese Biobank Study. Public Health. 2012;126:210–3. 10.1016/j.puhe.2011.11.012 22325671 PMC4340568

[3] Tamashiro KL, Sakai RR, Shively CA, Karatsoreos IN, Reagan LP. Chronic stress, metabolism, and metabolic syndrome. Stress. 2011;14:468–74. 10.3109/10253890.2011.606341 21848434

[4] McEwen BS. Central effects of stress hormones in health and disease: understanding the protective and damaging effects of stress and stress mediators. Eur J Pharmacol. 2008;583:174–85. 10.1016/j.ejphar.2007.11.071 18282566 PMC2474765

[5] Björntorp P. Do stress reactions cause abdominal obesity and comorbidities? Obes Rev. 2001;2:73–86. 10.1046/j.1467-789x.2001.00027.x 12119665

[6] Yan YX, Dong J, Liu YQ, Yang XH, Li M, Shia G, Association of suboptimal health status and cardiovascular risk factors in urban Chinese workers. J Urban Health. 2012;89:329–38. 10.1007/s11524-011-9636-8 22203493 PMC3324604

[7] Monzillo LU, Hamdy O. Evaluation of insulin sensitivity in clinical practice and in research settings. Nutr Rev. 2003;61:397–412. 10.1301/nr.2003.dec.397-412 14968910

[8] Yan YX, Dong J, Zhang J, Liu F, Wang W, Zhang L, Polymorphisms in NR3C1 gene associated with risk of metabolic syndrome in a Chinese population. Endocrine. 2014;47:740–8. 10.1007/s12020-014-0324-9 25104270

[9] Kristensen TS, Hannerz H, Høgh A, Borg V. The Copenhagen Psychosocial Questionnaire—a tool for the assessment and improvement of the psychosocial work environment. Scand J Work Environ Health. 2005;31:438–49. 10.5271/sjweh.948 16425585

[10] Shang L, Liu P, Fan LB, Gu HK, Li J Psychometric properties of the Chinese version of Copenhagen Psychosocial Questionnaire. J Environ Occup Med. 2008;25:572–6 (in Chin).

[11] Yan YX, Dong J, Liu YQ, Zhang J, Song MS, He Y, Association of suboptimal health status with psychosocial stress, plasma cortisol and mRNA expression of glucocorticoid receptor α/β in lymphocyte. Stress. 2015;18:29–34. 10.3109/10253890.2014.999233 25518867

[12] Matthews DR, Hosker JP, Rudenski AS, Naylor BA, Treacher DF, Turner RC. Homeostasis model assessment: insulin resistance and beta-cell function from fasting plasma glucose and insulin concentrations in man. Diabetologia. 1985;28:412–9. 10.1007/BF00280883 3899825

[13] Burén J, Eriksson JW. Is insulin resistance caused by defects in insulin’s target cells or by a stressed mind? Diabetes Metab Res Rev. 2005;21(6):487–94. 10.1002/dmrr.567 15977304

[14] Ware WR. Psychological stress, insulin resistance, inflammation and the assessment of heart disease risk. Time for a paradigm shift? Med Hypotheses. 2008;71(1):45–52. 10.1016/j.mehy.2008.02.008 18406066

[15] Eller NH, Netterstrøm B, Hansen AM. Psychosocial factors at home and at work and levels of salivary cortisol. Biol Psychol. 2006;73(3):280–7. 10.1016/j.biopsycho.2006.05.003 16824664

[16] Binnert C, Ruchat S, Nicod N, Tappy L. Dexamethasone-induced insulin resistance shows no gender difference in healthy humans. Diabetes Metab. 2004;30(4):321–6. 10.1016/S1262-3636(07)70123-4 15525874

[17] Nicod N, Giusti V, Besse C, Tappy L. Metabolic adaptations to dexamethasone-induced insulin resistance in healthy volunteers. Obes Res. 2003;11:625–31. 10.1038/oby.2003.90 12740452

[18] Sjöstrand M, Eriksson JW. Neuroendocrine mechanisms in insulin resistance. Mol Cell Endocrinol. 2009;297:104–11. 10.1016/j.mce.2008.05.010 18599191

[19] Lundgren M, Burén J, Ruge T, Myrnäs T, Eriksson JW. Glucocorticoids down-regulate glucose uptake capacity and insulin-signaling proteins in omental but not subcutaneous human adipocytes. J Clin Endocrinol Metab. 2004;89:2989–97. 10.1210/jc.2003-031157 15181089

[20] Barthel A, Scherbaum WA, Bornstein SR. [Novel aspects in the mechanisms of steroid diabetes and the regulation of hepatic glucose production by insulin and steroids]. Med Klin (Munich). 2003;98:283–6. 10.1007/s00063-003-1258-9 12721674

[21] Novelli M, Pocai A, Chiellini C, Maffei M, Masiello P. Free fatty acids as mediators of adaptive compensatory responses to insulin resistance in dexamethasone-treated rats. Diabetes Metab Res Rev. 2008;24:155–64. 10.1002/dmrr.785 18058844

